# A hospital-wide evaluation of delirium prevalence and outcomes in acute care patients - a cohort study

**DOI:** 10.1186/s12913-018-3345-x

**Published:** 2018-07-13

**Authors:** Maria Schubert, Roger Schürch, Soenke Boettger, David Garcia Nuñez, Urs Schwarz, Dominique Bettex, Josef Jenewein, Jasmina Bogdanovic, Marina Lynne Staehli, Rebecca Spirig, Alain Rudiger

**Affiliations:** 1 Nursing Science, Faculty of Medicine, Department of Public Health, University of Basel, Bernoullistr. 28, 4056 Basel, Switzerland; 2 Directorate of Nursing/MTT, Insel Gruppe, University Hospital Inselspital, Bern, Freiburgstr. 44a, 3010 Bern, Switzerland; 3 School of Health Professions, Institute of Nursing, Zurich University of Applied Science, Technikumstr. 81, P.O. Box, 8401, Winterthur, Switzerland; 4 Clinical Trial Unit, Institute of Social and Preventive Medicine, University of Bern, Finkenhubelweg 11, 3012 Bern, Switzerland; 5 Virginia Tech, Department of Entomology (MC0319), 170 Drillfield Drive, Blacksburg, VA 24061 USA; 6 Department of Psychiatry and Psychotherapy, University Hospital Zurich, Raemistr. 100, 8091 Zurich, Switzerland; 7 Center for Gender Variance, University Hospital Basel, Spitalstrasse 21, 4056 Basel, Switzerland; 8 Division of Neurology, University Hospital Zurich, Raemistr. 100, 8091 Zurich, Switzerland; 9 Institute of Anesthesiology, University of Zurich and University Hospital Zurich, Raemistrasse 100, 8091 Zurich, Switzerland; 10 Nursing Department, Balgrist University Hospital, Forchstrasse 340, 8008 Zurich, Switzerland; 11 Nursing and Allied Health Care Professions Office, University Hospital Zurich, Raemistr. 100, 8091 Zurich, Switzerland

**Keywords:** Delirium, Neurocognitive disorders, Hospital mortality, Length of stay, Cost of diseases OR economic burden of diseases

## Abstract

**Background:**

Delirium is a well-known complication in cardiac surgery and intensive care unit (ICU) patients. However, in many other settings its prevalence and clinical consequences are understudied. The aims of this study were: (1) To assess delirium prevalence in a large, diverse cohort of acute care patients classified as either at risk or not at risk for delirium; (2) To compare these two groups according to defined indicators; and (3) To compare delirious with non-delirious patients regarding hospital mortality, ICU and hospital length of stay, nursing hours and cost per case.

**Methods:**

This cohort study was performed in a Swiss university hospital following implementation of a delirium management guideline. After excluding patients aged < 18 years or with a length of stay (LOS) < 1 day, 29′278 patients hospitalized in the study hospital in 2014 were included.

Delirium period prevalence was calculated based on a Delirium Observation Scale (DOS) score ≥ 3 and / or Intensive Care Delirium Screening Checklist (ICDSC) scores ≥4.

**Results:**

Of 10′906 patients admitted, DOS / ICDSC scores indicated delirium in 28.4%. Delirium was most prevalent (36.2–40.5%) in cardiac surgery, neurosurgery, trauma, radiotherapy and neurology patients. It was also common in geriatrics, internal medicine, visceral surgery, reconstructive plastic surgery and cranio-maxillo-facial surgery patients (prevalence 21.6–28.6%). In the unadjusted and adjusted models, delirious patients had a significantly higher risk of inpatient mortality, stayed significantly longer in the ICU and hospital, needed significantly more nursing hours and generated significantly higher costs per case. For the seven most common ICD-10 diagnoses, each diagnostic group’s delirious patients had worse outcomes compared to those with no delirium.

**Conclusions:**

The results indicate a high number of patients at risk for delirium, with high delirium prevalence across all patient groups. Delirious patients showed significantly worse clinical outcomes and generated higher costs. Subgroup analyses highlighted striking variations in delirium period-prevalence across patient groups. Due to the high prevalence of delirium in patients treated in care centers for radiotherapy, visceral surgery, reconstructive plastic surgery, cranio-maxillofacial surgery and oral surgery, it is recommended to expand the current focus of delirium management to these patient groups.

**Electronic supplementary material:**

The online version of this article (10.1186/s12913-018-3345-x) contains supplementary material, which is available to authorized users.

## Background

Delirium is a sudden acute mental change accompanying acute illness. Characterized by disturbances of consciousness, attention, cognition, psychomotor behavior and emotions [1, 2], it affects 10 to 60% of all patients treated in medical, surgical, medical-surgical mixed or general wards [3–5], and up to 80% of those treated in intensive care units (ICUs) [3, 6–10]. More than two dozen predisposing and precipitating delirium risk factors have been identified, including male gender, older age (> 65 years), prior delirium, co-morbidities (i.e., dementia, depression) and severe illness [3, 11–13].

Delirium is linked to negative patient and institutional outcomes including prolonged ICU and hospital length of stay (LOS) [7, 14–17], higher mortality rates [14, 16, 18–20], cognitive decline or impaired cognitive functions [20–23], restrictions in motor functionality [23, 24], ongoing need for care in long-term care institutions [14, 15] and a higher likelihood of discharge to destinations other than home [23, 25].

From an economic perspective, delirium is strongly associated with additional healthcare costs [17]. In the United States (US), annual additional delirium-related healthcare costs are estimated to range from 6.6 to 20.4 billion USD (mean: 9014 USD per case) in ICU patients [26] and 38 to 152 billion USD per year in non-ICU patients aged 70 years and older (range: 16,303 to 64,421 USD per case) [27].

The large number of patients affected by delirium during hospitalization, the negative clinical outcomes, and the severe economic consequences all call for action. In recent years, several delirium management guidelines and / or standardized programs were developed for the prevention, early recognition, and / or treatment of delirium across all hospital departments [28–32]. The results indicate that multicomponent delirium management guidelines or programs are most efficient to reduce the delirium rates and the delirium-linked negative outcomes, i.e., decreasing LOS and institutionalization [31, 33, 34].

In 2012, in response to the delirium burden at the study hospital –a Swiss university hospital–the multi-professional Delir-Path (**D**etect **E**va**l**uate Control **I**npatient **R**isk factors, **P**revent **A**nd **T**reat **H**ospital Acquired Deliriums) project was started. It includes a practice development part and a health service research (HSR) part.

Delir-Path has five primary purposes: 1) to develop a standardized multi-professional, multicomponent delirium management guideline for the prevention, early recognition and treatment of delirium; 2) to implement the delirium management guideline throughout the study hospital and to monitor and evaluate the implementation process based on defined outcomes; 3) to evaluate the effectiveness, efficiency, and benefits of the implemented multiprofessional delirium management protocol, both as a whole and as single components, e.g., pharmacological delirium treatment; 4) to develop and implement a system for monitoring delirium incidence rates and the courses of the underlying diseases (duration); 5) to develop and establish a multicenter, multiprofessional Health Care Service Research Program for effective, efficient and cost-effective delirium management.

As a first step in a form of a pilot study an interprofessional multicomponent delirium management guideline was developed. The developed guideline contains interventions for delirium prevention, risk stratification, screening and diagnostics, non-pharmacological and pharmacological treatment, as well as a multi-professional training program (see Additional file 1). The guideline was implemented on nine surgical and neurological wards and ICUs and its benefits evaluated [35, 36]. Based on that pilot’s promising results, the standardized delirium management guideline was adapted and implemented hospital-wide in 2013 / 2014 [35, 36]. Concurrently, the HSR program was developed further. In the study hospital, as of April 2018, this includes a main study, more than 10 sub-studies [6, 36–43]. Furthermore, the program includes a multicenter study in ICU settings. This study refers to the main study, which uses a longitudinal cohort design.

To date, delirium management programs and / or delirium bundles and related research have focused on the best-known high delirium risk patient groups – predominately ICU, cardiac and orthopaedic patients [3]. A small number have also focused on medical and / are palliative care groups [44, 45]. However, the literature shows no delirium management programs implemented, e.g., for haemato-oncological patients, although, based on the typical courses of their conditions, fulfil the criteria for a high delirium risk [46].

Because significant risk groups may remain undetected, the Delir-Path main study included an entire university hospital cohort of acute care patients. This allowed us to study both delirium’s prevalence and the effect of the implemented guideline across acute care patient groups.

## Methods

### Aims

The aims of this study were: (1) to assess delirium prevalence in a cohort of acute care patients classified as either at risk or not at risk for delirium; (2) to compare these two groups according to defined indicators, including main diagnosis, care center, and admission and discharge details; and (3) to compare delirious with non-delirious patients regarding hospital mortality, ICU and hospital LOS, nursing hours and cost per case.

### Study design and setting

**Fig. 1:**
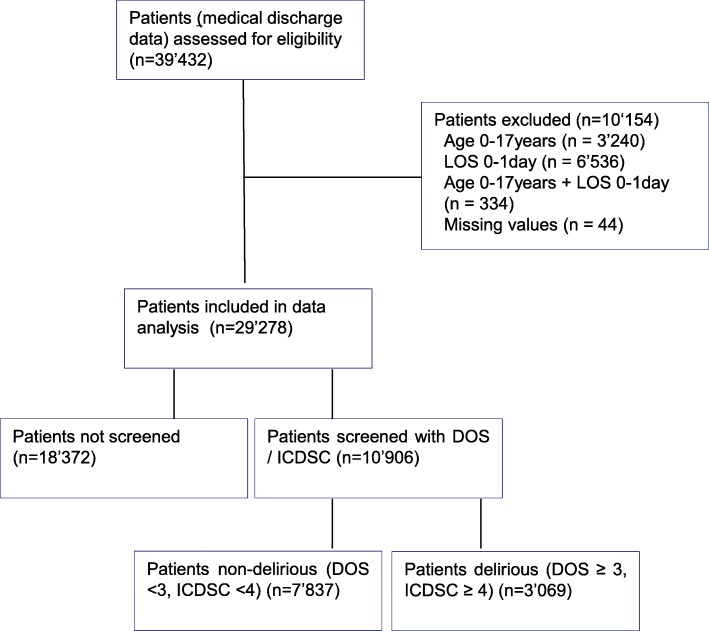
Sample and sampling per intervention group of patients hospitalized in 2014. Legend: DOS (Delirium Observation Scale); ICDSC (Intensive Care Delirium Screening Checklist); LOS (Length of Stay)

This study used data from the ongoing Delir-Path health service research program [6, 36–43] conducted in a Swiss university hospital with 800 beds distributed across 43 departments and institutes. As a longitudinal cohort study, it includes data of all eligible patients hospitalized in the study hospital between January and December 2014, 1 year after the implementation of the delirium guideline. Patients younger than 18 years of age, and those hospitalized for less than 1 day, were excluded (Fig. 1).

### Variables, measurements, data sources, data collection

#### Screening for and definition of delirium

In accordance with the implemented delirium management guideline, all patients admitted to the regular wards were screened for delirium once per shift with the Delirium Observation Scale (DOS) [47] for at least 3 days, if they fulfilled the following criteria: a) ≥ 65 years; or b) < 65 years with delirious symptoms, e.g., conspicuous symptoms such as disorientation or agitation. If the DOS Score indicated no delirium development for three consecutive days, the screening was stopped. If the patients developed a delirium, the screening was continued until the DOS score fell below 3 (see Additional file 1). All ICU patients with states of consciousness allowing assessment (Richmond Agitation Scale Score (RASS) [48] of − 3 to + 4) are screened once per shift with the Intensive Care Delirium Screening Checklist (ICDSC) [49] (see Additional file 1).

The DOS is a 13-item screening tool for non-ICU patients. It was developed to facilitate early recognition of delirium according to the criteria of the Diagnostic and Statistical Manual IV-TR. A DOS score ≥ 3 indicates a delirium. The DOS’s sensitivity (82, 89%) and specificity (86, 96%), as well as its reliability and validity were confirmed in several studies [47, 50, 51].

The ICDSC is an 8-item screening tool developed to detect delirium in ICU patients, with scores ≥4 indicating delirium [49]. In several studies, the tool has shown good sensitivity (64, 89, 99%), specificity (57, 64, 95%) and reliability (kappa 0.67, 0.91, 0.92) [38, 49, 52].

For the current study, a patient was considered delirious if he/she had at least once ICDSC score of ≥4 and / or a DOS score ≥ 3.

#### Outcomes

To describe the entire sample and to compare patients with and without delirium across patient subgroups, the following five main outcomes were selected: (1) Mortality, i.e., the frequency of patient deaths during hospitalization; (2) ICU LOS; (3) hospital LOS; (4) nursing hours per case, i.e., the number of nursing hours spent per case for direct patient care, assessed once per shift; and (5) total cost per case.

Other outcomes of interest and variables used as patient descriptors or as control variables were age, gender, residency before admission to the hospital (e.g., home, hospital), type of hospital admission (emergency, other), care center (organizational unit: e.g. cardiac surgery, visceral surgery, internal medicine), principal diagnoses (diagnostic codes drawn from the 19 ICD-10 chapters, diagnoses other than delirium), comorbidities, the presence of one or more disorders (ICD- 10 codes) as defined by Quan et al., [53], length of ICU stay**,** discharge destination (the destination to which the patient was discharged from the hospital, i.e., home, other institution), and readmission rate.

All variables / data are documented regularly in the patient medical records. They refer to the following databases: the Swiss Federal Statistical Office (FSO) medical and administrative database [54], the Minimal Data Set–Intensive Care Unit (MDSi) [55], the patient performance classification “Leistungserfassung in der Pflege” (LEP) [56] database, and DOS and ICDSC delirium screening data. The LEP performance classification is used for uniform documentation of services in the healthcare sector, and can be linked with other assessment, classification and outcome data. Authorized hospital administrative staff extracted the required patient data and made them available to the researchers. The researchers had no information that could be used to identify the patients from whom they had been collected. The data sets were linked based on unique case identification numbers.

### Statistical methods

Patient characteristics for the entire sample and subgroups were described using means (with standard deviations), medians (with 25 and 75% quartiles), or counts (and percentages).

We calculated the period prevalence as the ratio of the number of patients diagnosed with delirium over a given period to the number of eligible individuals in the hospitalized population during that period, presented as a percentage. We compared overall inpatient mortality of delirious to non-delirious patients using generalized linear models of the binomial family with logit link function. We compared ICU and hospital length of stays of delirious and non-delirious patients using Cox proportional hazard models. Finally, we compared costs per case and nursing hours using quantile regression. In an additional step, we adjusted these differences in mortality, LOS, cost and nursing hours by controlling for age, gender, pre-admission residence type, type of admission (emergency or not), Charlson co-morbidity index [53], type of service, and ICU stay (yes/no). In screened at-risk patients older than 65 years of age, we also performed an ICD-10 diagnosis subgroup analysis investigating differences between patients who developed delirium and those who did not. Five models analogous to the overall patient data set were constructed (i.e., one for each outcome variable). Contrasts were calculated 2. The two-sided level of significance was set at *P* = 0.05. All statistical tasks were performed using the R Statistical Package v. 3.4.2. In R, we used the packages survival 2.41–3 for Cox proportional hazard models and quantreg 5.35 for quantile regressions. Additionally, we used lsmeans 2.27–61 to calculate subgroup contrasts between delirious and non-delirious patients for GLMs and Cox proportional hazard models. Subgroup contrasts were calculated manually for quantile models, and significance was calculated based on standard errors following [57, 58].

## Results

### Participants and their characteristics

Of the 39′432 patients hospitalized in the study hospital in 2014, 29′278 (74.2%) were identified as eligible for this study and included in the analysis (Fig. 1). Characteristics of the overall patient sample (patients with vs. without delirium risk, delirious vs. non-delirious patients) are described in Table 1.

**Table 1:** Characteristics of the patients: entire sample, patient at risk screened for a delirium, patient at risk with and without a delirious state

	Entire sample			Screened sample – patient at risk for a delirium	
Endpoints / Variables	Total	Not screened (not at risk for a delirium)	Screened (at risk for a delirium)	Non-delirious DOS < 3, ICDSC < 4	Delirious DOS ≥ 3, ICDSC ≥ 4
Total n (%)	29′278	18′372 (62.8)	10′906 (37.2)	7′837 (71.9)	3′069 (28.1)
Age [years] mean (SD)	54.7 (19.2)	47.0 (16.9)	67.6 (15.4)	67.3 (15.2)	68.4 (15.8)
Gender [male] n (%)	14′147 (48.3)	7′672 (41.8)	6′475 (59.4)	4′601 (58.7)	1′874 (61.1)
Residency prior admission n (%)					
Home	25′842 (88.3)	17′003 (92.5)	8′839 (81.0)	6′730 (85.9)	2′109 (68.7)
Other hospital	2′386 (8.1)	928 (5.1)	1'458 (13.4)	813 (10.4)	645 (21.0)
Nursing home	402 (1.4)	121 (0.7)	281 (2.6)	125 (1.6)	156 (5.1)
Other residency	462 (1.6)	245 (1.3)	217 (2.0)	99 (1.3)	118 (3.8)
With home care	186 (0.6)	75 (0.4)	111 (1.0)	70 (0.9)	41 (1.3)
Emergency admission n (%)	13′862 (47.3)	8′727 (47.5)	5′135 (47.1)	3′280 (41.9)	1′855 (60.4)
Type of care n (%)					
Internal / general medicine	7′198 (24.6)	4′218 (23.0)	2′980 (27.3)	2′141 (27.3)	839 (27.3)
Cardiac surgery	1′392 (4.8)	249 (1.4)	1′143 (10.5)	680 (8.7)	463 (15.1)
Neurology	1′516 (5.2)	517 (2.8)	999 (9.2)	637 (8.1)	362 (11.8)
Neurosurgery	968 (3.3)	173 (0.9)	795 (7.3)	480 (6.1)	315 (10.3)
Trauma surgery	2′090 (7.1)	1'360 (7.4)	730 (6.7)	462 (5.9)	268 (8.7)
Other service	16′114 (55.0)	11′855 (64.5)	4′259 (39.1)	3′437 (43.9)	822 (26.8)
ICD-10 chapter n (%)					
IX. Diseases of circulatory system	4′538 (15.5)	1′218 (6.6)	3′320 (30.4)	2′247 (28.7)	1′073 (35.0)
II. Neoplasms	5′665 (19.3)	3′182 (17.3)	2′483 (22.8)	1′915 (24.4)	568 (18.5)
XIX. Injury/poisoning of external cause	2′997 (10.2)	1′938 (10.5)	1′059 (9.7)	653 (8.3)	406 (13.2)
XI. Diseases of the digestive system	1′954 (6.7)	1'326 (7.2)	628 (5.8)	448 (5.7)	180 (5.9)
VI. Diseases of nervous system	933 (3.2)	399 (2.2)	534 (4.9)	359 (4.6)	175 (5.7)
I. Infections/parasites	728 (2.5)	492 (2.7)	236 (2.2)	120 (1.5)	116 (3.8)
X. Diseases of respiratory system	1'440 (4.9)	966 (5.3)	474 (4.3)	367 (4.7)	107 (3.5)
Other ICD-10 chapters	11,'023 (37.6)	8'851 (48.2)	2'172 (19.9)	1'728 (22.0)	444 (14.5)
ICU stay n (%)	4'002 (13.7	502 (2.7)	3'500 (32.1)	1'912 (24.4)	1'588 (51.7)
Discharged n (%)					
Home	23,'679 (80.9)	16′767 (91.3)	6′912 (63.4)	5′869 (74.9)	1′043 (34.0)
Rehab	2'584 (8.8)	521 (2.8)	2'063 (18.9)	1′106 (14.1)	957 (31.2)
Other hospital	929 (3.2)	219 (1.2)	710 (6.5)	343 (4.4)	367 (12.0)
Nursing home	599 (2.0)	190 (1.0)	409 (3.8)	172 (2.2)	237 (7.7)
Others service	266 (0.9)	120 (0.7)	146 (1.3)	57 (0.7)	89 (2.9)
Missing	503 (1.7)	279 (1.5)	224 (2.1)	131 (1.7)	93 (3.0)
Re-admissions n (%)	910 (3.1)	546 (3.0)	364 (3.3)	252 (3.2)	112 (3.6)
Outcomes					
Hospital mortality n (%)	718 (2.5)	276 (1.5)	442 (4.1)	159 (2.0)	283 (9.2)
LOS in the hospital d median [Q1, Q3]	5.00 [3, 10]	4 [3, 7]	8 [4, 15	7 [4, 12]	13 [7, 23],
Nursing hours per case h median [Q1, Q3]	30.1 [17.7, 59.5]	24.1 [15.7, 39.9]	52.5 [26.0, 107.7]	41.0 [22.2, 76.0]	114.5 [56.7, 240.5]
Cost per case CHF	11′128 [6′667, 22′861]	8′764 [5′788, 14′515]	20′875, [10′463, 42′271]	16′662 [9067, 32′413]	40′259 [19′235, 80′245]

Legend: *DOS* delirium observation scale; *ICDSC* intensive care delirium screening checklist; *ICU* intensive care unit

Whereas patients not at risk were generally admitted as elective (non-emergency) cases from home to the hospital, the screened patients, particularly those in delirious states, were more likely to have been transferred from another hospital and / or admitted via emergency admission. Post-admission, they were more likely to spend time in the ICU and more likely to be discharged to a rehabilitation center or another hospital (Table 1).

### Delirium prevalence in different patient subgroups (type of care, ICD-10 diagnosis)

In accordance with the implemented guideline, 10′906 (37.2%) of the total 29′278 patients were identified at risk for delirium and screened. Of the screened group, 3′069 (28.1%) yielded at least one DOS score ≥ 3 and / or ICDSC score ≥ 4, indicating a delirious state (Table 1). The number of delirious patients varied by type of care and ICD-10 diagnosis chapter. A delirious state was most frequent in internal / general medicine patients, followed, in descending order of prevalence, by cardiac surgery, neurology, neurosurgery and trauma surgery patients. This is partly reflected in the ICD-10 chapter groups, where delirium was more prevalent in patients with a principal diagnosis related to the ICD-10 chapters IX (“Diseases of circulatory system”), II (“Neoplasms”) and XIX (“Injury/poisoning of external cause”) (Table 1).

**Fig. 2:**
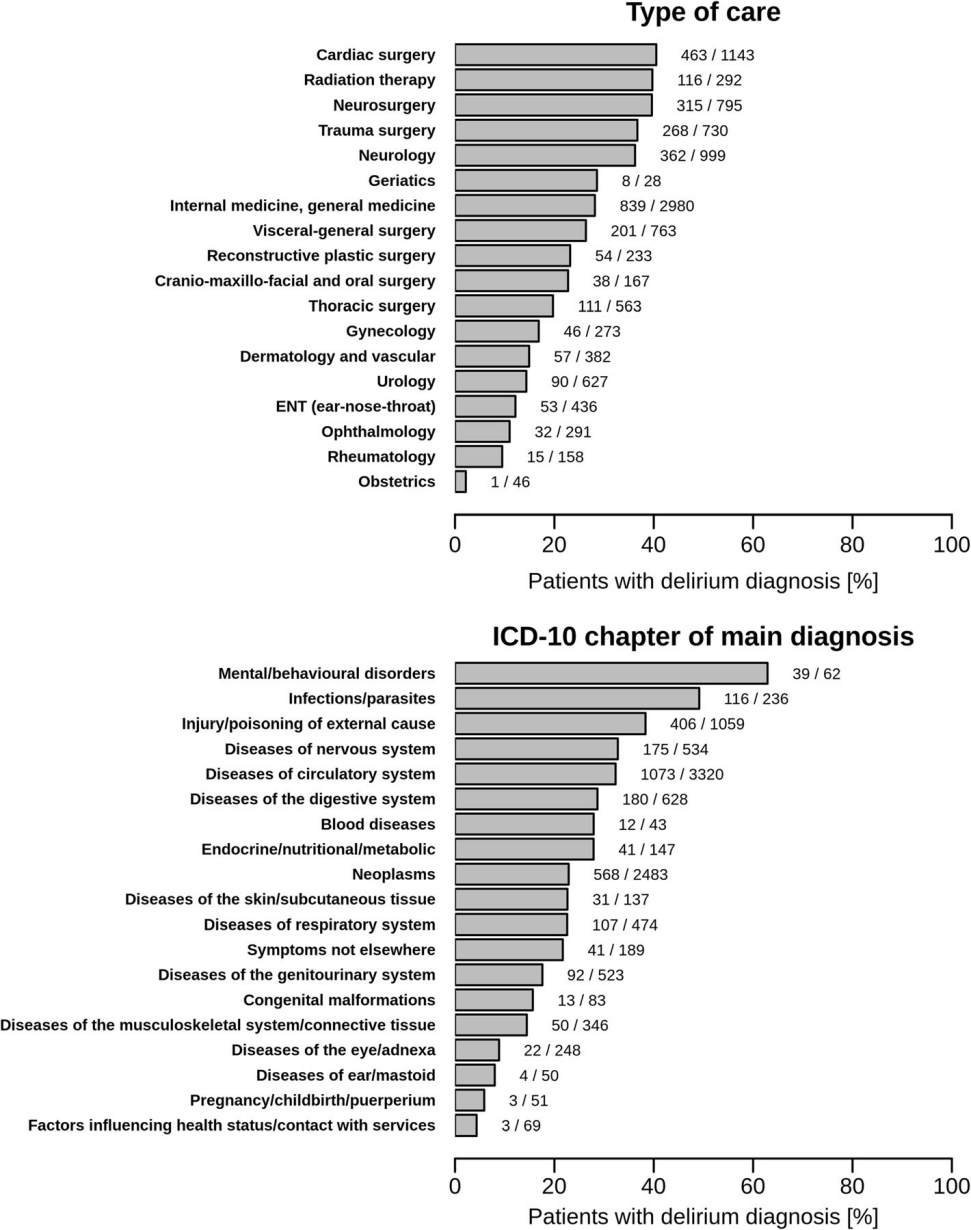
Percentage of patients diagnosed with delirium (DOS, ICDSC scores) within type of care and ICD-10 chapter of main diagnosis. Legend/notes: Patients with missing values were ignored when calculating percentages

The comparisons within and across the various care units and ICD-10 chapter groups (Fig. 2) show that a delirious state was most frequent in patients admitted to cardiac surgery units (40.5%), followed by radiotherapy (39.7%), neurosurgery (39.6%), trauma surgery (36.7%) and neurology (36.2%) care (Fig. 2). With reference to the ICD-10 chapters, delirium was most frequent in patients with a principal diagnosis related to group V (“Mental / behavioral disorders”) (62.9%), followed by groups I (“Infections/parasite diseases”) (49.2%), XIX (“Injury/poisoning/external causes”) (38.3%), VI (“Diseases of the nervous system”) (32.8%), IX (“Diseases of the circulatory system”) (32.3%), XI (“Diseases of the digestive system”) (28.7%), III (“Blood diseases”) (27.9%) and IV (Endocrine/nutritional/metabolic diseases) (27.9%) (Fig. 2).

### Mortality, ICU and hospital length of stay, mortality, cost and nursing hours per case in delirious and non-delirious patients

The comparison of the raw number of patients identified, in accordance with the implemented guideline, as not at risk for a delirium (*n* = 18′372) with those identified as at risk (*n* = 10′906) and screened shows that at-risk patients stayed twice as long in hospital, accounting for twice the number of nursing hours and twice the total cost per case. The comparison of the screened patients identified as not delirious (*n* = 7′837) versus delirious (*n* = 3′069) shows the same pattern (Table 1).

In screened patients 65 years or older, cases with a delirium were at a higher risk of dying (crude mortality OR (95% CI): 5.46 (4.85 to 6.15), *p* <  0.001), stayed longer at the ICU (crude HR (95% CI) for discharge: 0.40 (0.38 to 0.42), *p* <  0.001) and in the hospital (crude HR (95% CI) for discharge: 0.25 (0.24 to 0.26), *p* <  0.001), they caused more nursing hours (crude median difference nursing hours (95% CI): 64.8 (62.0 to 67.6), *p* <  0.001, and they cost more (crude median cost difference [in thousand CHF] (95% CI): 20.9 (19.9 to 21.9), < 0.001). If we adjusted for age, gender, pre-admission residence type, type of admission (emergency or not), Charlson co-morbidity index, type of service, and ICU stay, these differences were less pronounced, but still highly significant (adjusted mortality OR (95% CI): 3.18 (2.79 to 3.63), *p* <  0.001; adjusted HR (95% CI) for ICU discharge: 0.39 (0.36 to 0.41), *p* <  0.001; adjusted HR (95% CI) for hospital discharge: 0.43 (0.41 to 0.45), *p* <  0.001; adjusted median difference nursing hours (95% CI): 37.3 (35.0 to 39.5), *p* <  0.001; adjusted median cost difference [in thousand CHF] (95% CI): 10.0 (9.3 to 10.6), < 0.001).

**Fig. 3:**
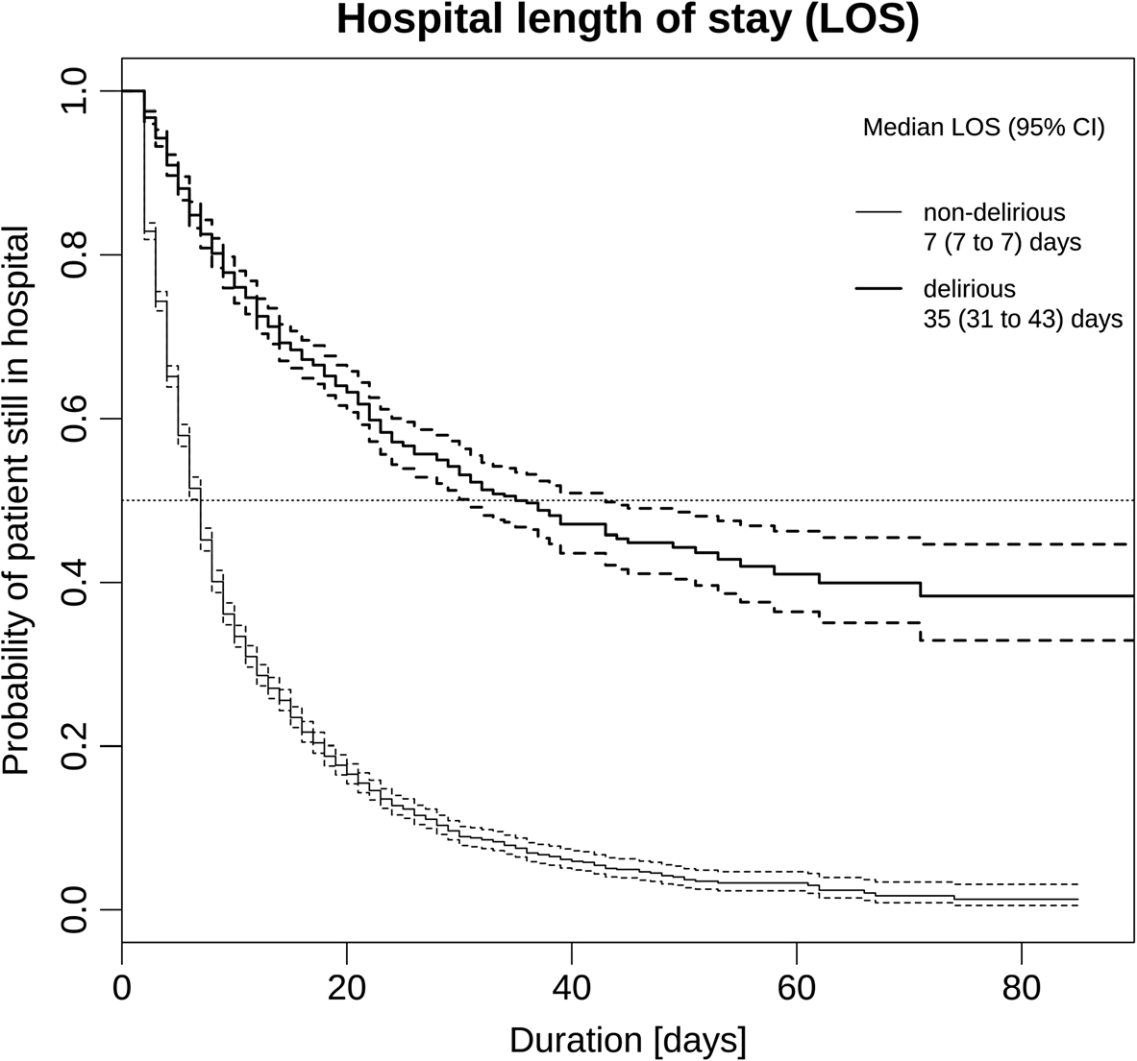
Duration of the hospital length of stay in delirious and non-delirious patients

In our further exploration of the differences between non-delirious and delirious patients, only patients ≥65 years who were judged at risk and screened for a delirium (*n* = 7′446) were included. Of these, 2′057 (38%) were identified as delirious. (Table 2). For a comparison between the non-delirious and the delirious patients, a set of non-adjusted outcome models were fit across seven ICD-10 diagnoses groups with reference to the five selected outcomes (Table 2). Compared to non-delirious patients, those who were delirious were significantly more likely to die (3–11 times). They were also significantly less likely to be discharged from the ICU (1–9 times) or hospital (3–9 times). This applied to all patients except those with infectious diseases and diseases of the respiratory system: in their cases, the differences were not significant. Delirious patients also required significantly more nursing hours (1.5–4 times) and generated significantly higher costs per case (1.5 to 3.5 times) (Table 2). Differences in overall hospital LOS for screened patients with versus without delirium are displayed in Fig. 3.

**Table 2:** In-hospital mortality, LOS in the ICU and in hospital, nursing hours and costs per case in delirious and non-delirious patients with a main diagnosis, which refers to the top-seven ICD-10 diagnoses chapters

Outcome by ICD-10 chapter		Non-delirious (DOS < 3, ICDSC < 4)		Delirious (DOS ≥ 3, ICDSC ≥4)	Difference	*p*
Mortality	*n*	*n dead (%)*	*n*	*n dead (%)*	*odds ratio (95% CI)*	
Diseases of circulatory system	1'470	41 (3%)	758	67 (9%)	3.38 (2.27 to 5.04)	< 0.001
Neoplasms	1'295	36 (3%)	380	51 (13%)	5.42 (3.48 to 8.45)	< 0.001
Injury/poisoning of external cause	446	12 (3%)	256	28 (11%)	4.44 (2.22 to 8.90)	< 0.001
Diseases of the digestive system	315	5 (2%)	101	15 (15%)	10.81 (3.82 to 30.59)	< 0.001
Diseases of nervous system	242	3 (1%)	113	7 (6%)	5.26 (1.33 to 20.74)	0.0177
Infections/parasites	80	5 (6%)	78	22 (28%)	5.89 (2.10 to 16.52)	< 0.001
Diseases of respiratory system	232	7 (3%)	59	12 (20%)	8.21 (3.07 to 21.95)	< 0.001
other	1'214	6 (0%)	253	14 (6%)	11.79 (4.49 to 30.98)	< 0.001
ICU LOS [days]	*n*	*median (95% CI)*	*n*	*median (95% CI)*	*hazard ratio (95% CI)*	
Diseases of circulatory system	331	1.0 (0.9 to 1.0)	384	3.1 (2.7 to 4.0)	0.43 (0.37 to 0.50)	< 0.001
Neoplasms	174	0.9 (0.9 to 0.9)	147	2.9 (1.9 to 4.5)	0.37 (0.29 to 0.46)	< 0.001
Injury/poisoning of external cause	61	0.9 (0.8 to 1.4)	133	3.7 (3.0 to 5.4)	0.47 (0.34 to 0.64)	< 0.001
Diseases of the digestive system	40	1.0 (0.9 to 1.7)	47	10.8 (4.6 to 13.5)	0.28 (0.18 to 0.44)	< 0.001
Diseases of nervous system	41	0.8 (0.7 to 0.8)	38	1.8 (0.8 to 7.5)	0.26 (0.16 to 0.40)	< 0.001
Infections/parasites	8	2.0 (0.9 to –)	29	13.5 (5.2 to 25.1)	0.65 (0.22 to 1.90)	0.434
Diseases of respiratory system	33	1.1 (1.0 to 2.0)	17	1.8 (1.0 to 15.0)	0.64 (0.34 to 1.17)	0.148
other	74	0.9 (0.9 to 1.5)	56	4.9 (3.9 to 11.0)	0.34 (0.24 to 0.49)	< 0.001
Hospital LOS [days]	*n*	*median (95% CI)*	*n*	*median (95% CI)*	*hazard ratio (95% CI)*	
Diseases of circulatory system	1'470	8 (8 to 9)	758	71 (43 to –)	0.21 (0.18 to 0.25)	< 0.001
Neoplasms	1'295	7 (6 to 7)	380	26 (22 to 37)	0.30 (0.25 to 0.35)	< 0.001
Injury/poisoning of external cause	446	8 (7 to 9)	256	37 (33 to –)	0.23 (0.17 to 0.30)	< 0.001
Diseases of the digestive system	315	6 (6 to 7)	101	31 (21 to –)	0.26 (0.19 to 0.36)	< 0.001
Diseases of nervous system	242	7 (6 to 7)	113	25 (15 to –)	0.31 (0.23 to 0.43)	< 0.001
Infections/parasites	80	7 (6 to 12)	78	58 (43 to –)	0.18 (0.11 to 0.30)	< 0.001
Diseases of respiratory system	232	8 (7 to 10)	59	17 (14 to –)	0.42 (0.27 to 0.65)	< 0.001
other	1'309	5 (5 to 6)	312	19 (15 to 24)	0.32 (0.27 to 0.38)	< 0.001
Nursing hours [in hours]	*n*	*median (25%; 75% quantiles)*	*n*	*median (25%; 75% quantiles)*	*median difference (95% CI)*	
Diseases of circulatory system	1'470	39.9 (20.3; 73.4)	758	119.2 (63.5; 226.4)	79.2 (70.1 to 88.4)	< 0.001
Neoplasms	1'295	36.9 (21.3; 67.7)	380	101.2 (42.1; 192.6)	63.9 (48.3 to 79.5)	< 0.001
Injury/poisoning of external cause	446	44.1 (24.8; 74.5)	256	102.9 (51.5; 195.8)	58.8 (42.2 to 75.5)	< 0.001
Diseases of the digestive system	315	28.9 (19.6; 57.8)	101	100.2 (37.2; 326.3)	71.3 (25.1 to 117.5)	0.00255
Diseases of nervous system	242	27.9 (18.5; 45.5)	113	71.7 (37.6; 122.7)	43.9 (28.4 to 59.5)	< 0.001
Infections/parasites	80	31.8 (17.2; 67.2)	78	128.2 (62.9; 298.3)	95.8 (74.1 to 117.6)	< 0.001
Diseases of respiratory system	232	38.8 (19.4; 73.6)	59	66.5 (41.6; 120.3)	27.8 (18.2 to 37.3)	< 0.001
other	1'309	25.1 (15.9; 46.1)	312	62.6 (29.0; 142.0)	37.6 (27.6 to 47.6)	< 0.001
Cost [in thousand CHF]	*n*	*median (25%; 75% quantiles)*	*n*	*median (25%; 75% quantiles)*	*median difference (95% CI)*	
Diseases of circulatory system	1'470	20.4 (11.6; 40.6)	758	48.3 (23.3; 79.7)	27.7 (23.0 to 32.4)	< 0.001
Neoplasms	1'295	13.7 (7.2; 27.4)	380	35.3 (15.4; 63.7)	21.5 (17.8 to 25.3)	< 0.001
Injury/poisoning of external cause	446	15.2 (9.0; 25.2)	256	34.9 (19.6; 67.7)	19.5 (15.6 to 23.4)	< 0.001
Diseases of the digestive system	315	11.3 (6.8; 19.8)	101	31.0 (13.0; 90.0)	19.7 (9.7 to 29.7)	< 0.001
Diseases of nervous system	242	10.9 (7.8; 18.3)	113	20.6 (13.5; 44.0)	9.8 (6.6 to 13.0)	< 0.001
Infections/parasites	80	9.9 (6.1; 19.0)	78	35.4 (16.6; 97.4)	24.4 (18.1 to 30.7)	< 0.001
Diseases of respiratory system	232	12.4 (7.6; 24.8)	59	18.8 (13.1; 29.8)	6.5 (4.2 to 8.7)	< 0.001
other	1'214	9.2 (6.0; 15.9)	253	16.6 (9.0; 32.6)	7.4 (4.7 to 10.1)	< 0.001

Legend: *ICU* intensive care unit; *LOS* length of stay, missing values for LOS due to censoring are indicated with a dash (−),

## Discussion

Of all eligible adult patients hospitalized in 2014, almost a third was identified as at risk for delirium and screened accordingly. Of those screened, nearly one-third yielded a DOS and / or ICDSC score indicating a delirium. In accordance with available evidence, delirium was more frequent in males and patients hospitalized for cardiac surgery [3, 6, 59, 60], neurosurgery, traumatology, radiotherapy, and neurological care, with period-prevalences from 36.2 to 40.5%. Elsewhere, with period-prevalence’s ranging from 21.6 to 28.6%, delirium also occurred frequently in patients treated in geriatric and internal / general medicine, as well as in visceral surgery. Similar prevalences were found in patients recovering from visceral surgery, reconstructive plastic surgery, cranio-maxillo-facial surgery, and oral surgery. This new finding underscores the importance of a standard delirium management guideline for use across patient groups.

The comparison of the delirium point-prevalence rate by ICD-10 diagnosis chapter and the presence of delirium in patients within and between groups partly reflected these rates. Delirious states were most frequent in patients with main diagnoses referring to the ICD-10 chapters on mental / behavioral disorders, infections, injuries, poisoning, or diseases of the nervous, circulatory, digestive, blood and endocrine /nutritional /metabolic systems. The high prevalence of delirious states in patients with diagnoses related to Chapter V (mental / ‘behavioral disorders) can be explained by that chapter’s direct focus on psychiatric disorders. The other diagnosis chapters refer to diseases such as infections or abnormal laboratory values, e.g., abnormal creatinine, serum sodium, glucose or potassium levels and metabolic acidosis, which are known precipitating factors of delirium [11, 61, 62].

Our subgroup analysis by ICD-10 diagnosis chapter indicated not only that delirious patients were significantly more likely to die, but that they required roughly twice most non-delirious groups’ nursing hours, ICU days, hospital stay, and overall financial expenditure per case. The one exception was the category of non-delirious patients with infections and respiratory diseases, whose ICU stays were not significantly lower (Table 2). These demonstrated negative outcomes in patients with delirium correspond with recent findings of significant differences between delirious and non-delirious patients’ mortality rates [14, 19, 63–65], ICU LOS [14, 16, 19, 66, 67], hospital LOS [14, 16, 17, 19, 66, 67], nursing hours per case [68] and total cost [17, 26, 67]. The demonstrated variation in these outcomes regarding the seven top ICD-10 diagnosis groups adds to the current evidence. Such marked differences between main outcomes suggest that group-specific characteristics and risk factors influence delirium-related outcomes and consequences. Still, it remains unclear whether delirium contributes to poorer outcomes, impacts the underlying illness’s trajectory [69], or simply indicates greater disease severity.

As our results show, compared to non-delirious patients, those with delirium were more often admitted from other hospitals and less frequently discharged home. In addition, they were treated in the ICU more frequently (table1). This suggests that, compared to their non-delirious counterparts, the delirious were more seriously ill or had more exacerbated functional health status impairment. Since serious disease and functional impairment are known predictors of delirium [3, 11, 12], despite controlling for all known confounders in the first set of models, which we run for the comparison of the non-delirious and delirious patients, we cannot exclude the possibility that these factors contributed to the outcome differences discussed above.

This study has a number of notable strengths and limitations. One strength is that its analyses covered all eligible patients treated in the study hospital over one full calendar year. The sample included a broad range of surgical, medical, and mixed surgical-medical patient groups – several of which, to our knowledge, were studied for the first time in this context. Two analyses were particularly important: that of the patient subgroup identified as at risk for delirium; and, within this group, the separate analysis of only the older at-risk patients by ICD-10 main diagnosis chapter. Both provided important insights regarding the occurrence of delirium in diverse subgroups. Limitations include an observational design and the inclusion of only a single center, both of which limit the generalizability of the results and allow no inferences regarding causal relations. Another limitation is that our chosen approach to identifying delirious patients – classing a patient as delirious if he/she had at least once an DOS score ≥ 3 and / or a ICDSC score of ≥4 - might have been too sensitive, i.e., it might have led to delirium rate overestimation. Further, the use of existing clinical data restricted both the number of variables and the overall adequacy of the dataset regarding our needs. This and the large sample size, for example, made a precise calculation of delirium intensity and duration unfeasible. Finally, in the study hospital, roughly 2′000 clinicians participated in delirium screening. Although these clinicians’ delirium training covered interrater reliability, a smaller research team may have yielded more reliable assessment results. I.e., while the use of validated screening tools ensured that detection of patients with delirium was reliable within acceptable tolerances. Further studies, ideally of a prospective nature, will be necessary to confirm our findings.

## Conclusions

The results reported here indicate not only high numbers of patients identified as at risk for a delirium but also high delirium prevalence across most patient groups. This underscores the relevance of this topic and the need for systematic delirium management to prevent, recognize, and treat delirium across patient groups.

In both the unadjusted and adjusted models, compared to the patients without delirium, delirious patients’ ≥ 65 year had a significantly higher mortality rate and longer ICU and hospital LOS. They also required more nursing hours and generated much higher cost per case. In the subgroup analysis conducted by ICD-10 diagnosis chapter, for the unadjusted models, each diagnostic group’s delirious patients had worse outcomes compared to those with no delirium. However, considering that, as discussed above, the current study cannot indicate causality, is it essential to test these findings with a study design that allows causal inferences.

Our subgroup analyses highlighted striking variations in delirium period-prevalence across patient groups. Several of these differences, e.g., the frequent occurrence of delirium in cardiac surgery or geriatric patients, support previous findings. One new finding – which requires further attention – is the high prevalence of delirium in radiotherapy, visceral surgery, reconstructive plastic surgery, and cranio-maxillo-facial or oral surgery patients – all groups who have thus far fallen either outside or only marginally within the scope of clinical delirium management.

In the subgroup analysis according to the ICD-10 diagnosis chapters, patient diagnoses referring to known delirium precipitating factors, e.g., infection/parasites, injury, poisoning, or endocrine /nutritional /metabolic diseases, were linked with elevated delirium prevalence. Therefore, in addition to patients currently in the focus of standard delirium management (i.e., ICU, cardiac and orthopaedic patients), it is recommended to expand that focus to include those treated in care centers for radiotherapy, visceral surgery, reconstructive plastic surgery, cranio-maxillo-facial and oral surgery.

Finally, the used approach described above to identify patients at risk for a delirium, timely screening, detection and identification of delirious patients can easily be added to standard clinical practice. From a scientific perspective, this approach allows quick classification of patients into three groups with significant and clinically relevant outcomes: (1) those not at risk, for whom delirium screening is unnecessary; (2) those at risk and screened, who do not develop a delirious state; and (3) those at risk and screened and who develop a delirious state.

## Additional file

Additional file 1: Components of the delirium management protocol. (PDF 101 kb)
