# Supplementary material for: A hospital-wide evaluation of delirium prevalence and outcomes in acute care patients - a cohort study
Source: BMC Health Serv Res. 2018 Jul 13;18:550. doi: 10.1186/s12913-018-3345-x (PMC6045819; doi:10.1186/s12913-018-3345-x)
Supplement: Supplementary file 1 — Components of the delirium management protocol. (PDF 101 kb) [file 12913_2018_3345_MOESM1_ESM.pdf]

1 Components of the delirium management protocol

**Delirium prevention**:	Based on individual patient risk factor constellations and 13 defined delirium risk areas, nurses and physicians select and conduct appropriate delirium prevention measures. Delirium risk control includes the prevention /correction of electrolytic disturbances and dehydration, improvement of communication and orientation with clocks and calendars, regular verbal communication with the patient, avoidance of immobilization; multiprofessional non-pharmacological and pharmacological delirium prevention measures are selected [1].
**Early recognition of deliriums**	All patients admitted who were aged ≥ 65 or regular ward patients of any age, showing conspicuous symptoms such as disorientation or agitation are screened for delirium once per shift at their bedside with the Delirium Observation Scale (DOS) [2] for at least three days. A DOS score ≥ 3 indicates a delirium. If DOS scores are unremarkable, screening is stopped after three days. If a patient develops delirium, the screening is continued until the DOS score falls below 3.<br>The DOS is a 13-item screening tool for non-ICU patients. It facilitates early recognition of delirium according to Diagnostic and Statistical Manual-IV criteria. In several studies the sensitivity (82-89%) and specificity (86-96%), as well as the reliability and validity of the tool have been confirmed [2, 3].<br>Once per shift, all Intensive Care Unit (ICU), Intermediate Care (IMC), and Post-Anesthesia Care Unit (PACU) patients whose consciousness allows a screening (Richmond Agitation Sedation Scores (RASS) -3 to +4) are screened for delirium by the bedside nurse via the Intensive Care Delirium Screening Checklist (ICDSC) [4]. An ICDSC score ≥ 4 indicates a delirium.<br>The ICDSC is an 8-item screening tool developed for delirium detection in ICU patients [4]. In several studies, the tool has shown good sensitivity (64%, 89%, 99%) and specificity (57%, 64%, 95%), as well as good validity and reliability (kappa 0.67, 0.91, 0.92) [4, 5].
**Delirium diagnostic**	For regular ward patients, at the first DOS Score ≥ 3, more specific assessments are conducted with the Mental-Status-Questionnaire (MSQ) [6], the Monate Rückwärts Zählen (MRZ, “Bedsite Confusion Scale”) instrument [7]and the [8]Confusion Assessment Method (CAM). The MSQ is a 10-item tool to assess cognition and attention[6]; the MRZ used includes one item from the Bedsite Confusion Scale (BCS); the BCS was developed by Stillman & Rybicki, 2000 to detect alteration in attention[7]; and the CAM is a 4-item diagnostic tool developed by Inouye (1990) to identify delirium based on defined criteria [8]. The tool is frequently used and shows good sensitivity (81%, 94% - 100%) and specificity (63%, 89% 90%-95%), as well as good reliability and validity [8, 9].<br>In all patients (regular ward, ICU, IMC, PACU) with screening scores indicating a delirium (ICDSC Score ≥ 4, positive CAM score and / or DOS

	Score ≥ 3), physicians and nurses conduct further delirium diagnostic tests. This includes defined laboratory tests and other specific tests to confirm / exclude the initially indicated delirium and etiology (i.e., infection, pulmonary deterioration, metabolic disorders). In patients with a confirmed delirium, nurses and physicians document the appropriate medical and nursing diagnoses in the patient charts.
**Delirium treatment**:	All patients with confirmed delirium receive a pharmacological delirium treatment with pipamperon p.o. (hyperactive symptoms) or haloperidol p.o. (i.v. in ICU, IMC patients) (hypoactive symptoms) [10] and, if required, other medications to treat accompanying symptoms. E.g., if a patient with hypoactive symptoms also shows restlessness, a combination of both medications and / or lorazepam s.l. is applied. In non-cooperative delirious patients who do not respond to the previous pharmacological therapy, continuous dexmedetomidine, clonidine or midazolam infusions are used for a defined time.
**Training program for nurses and physicians**	To train the nurses and physicians in the different components of delirium management, as defined in the protocol, the multiprofessional project team has developed a training program (eLearning, face-to-face lectures).

References

1. Michaud, L., et al., Delirium: guidelines for general hospitals. J Psychosom Res, 2007. 62(3): p. 371-83; Inouye, S.K., et al., A multicomponent intervention to prevent delirium in hospitalized older patients. N Engl J Med, 1999. 340(9): p. 669-76.
2. Schuurmans, M.J., L.M. Shortridge-Baggett, and S.A. Duursma, The Delirium Observation Screening Scale: a screening instrument for delirium. Res Theory Nurs Pract, 2003. 17(1): p. 31-50.
3. Koster, S., et al., The delirium observation screening scale recognizes delirium early after cardiac surgery. Eur J Cardiovasc Nurs, 2009; Gemert van, L.A. and M.J. Schuurmans, The Neecham Confusion Scale and the Delirium Observation Screening Scale: capacity to discriminate and ease of use in clinical practice. BMC Nurs, 2007. 6: p. 3; Detroyer, E., et al., Detection of delirium in palliative care unit patients: A prospective descriptive study of the Delirium Observation Screening Scale administered by bedside nurses. Palliat Med, 2013.
4. Bergeron, N., et al., Intensive Care Delirium Screening Checklist: evaluation of a new screening tool. Intensive Care Med, 2001. 27(5): p. 859-64.
5. Ouimet, S., et al., Subsyndromal delirium in the ICU: evidence for a disease spectrum. Intensive Care Med, 2007. 33(6): p. 1007-13; Schubert, M., et al., Entwicklung eines interprofessionellen Behandlungskonzepts Delir für eine medizinische und eine chirurgische Intensivstation. Intensiv Fachzeitschrift für Intensivpflege und Anästhesie, 2010. 6(10): p. 316-323.
6. Kahn, R.L., et al., Brief objective measures for the determination of mental status in the aged. Am J Psychiatr, 1960. 117: p. 326-28.
7. Stillman, M.J. and L.A. Rybicki, The bedside confusion scale: development of a portable bedside test for confusion and its application to the palliative medicine population. J Palliat Med, 2000. 3(4): p. 449-56.
8. Inouye, S.K., et al., Clarifying confusion: the confusion assessment method. A new method for detection of delirium. Ann Intern Med, 1990. 113(12): p. 941-8.
9. Wei, L.A., et al., The Confusion Assessment Method: a systematic review of current usage. J Am Geriatr Soc, 2008. 56(5): p. 823-30.
10. Devlin, J.W., et al., Current perceptions and practices surrounding the recognition and treatment of delirium in the intensive care unit: a survey of 250 critical care pharmacists from eight states. Ann Pharmacother, 2011. 45(10): p. 1217-29; Devlin, J.W. and Y. Skrobik, Antipsychotics for the prevention and treatment of delirium in the intensive care unit: what is their role? Harv Rev Psychiatry, 2011. 19(2): p. 59-67.
